# A Systems Biology Approach Identifies Hidden Regulatory Connections Between the Circadian and Cell-Cycle Checkpoints

**DOI:** 10.3389/fphys.2020.00327

**Published:** 2020-04-16

**Authors:** Xianlin Zou, Dae Wook Kim, Tetsuya Gotoh, Jingjing Liu, Jae Kyoung Kim, Carla V. Finkielstein

**Affiliations:** ^1^Integrated Cellular Responses Laboratory, Department of Biological Sciences, Fralin Life Sciences Institute, Virginia Tech, Blacksburg, VA, United States; ^2^Department of Mathematical Sciences, Korea Advanced Institute of Science and Technology, Daejeon, South Korea

**Keywords:** circadian rhythms, tumor suppressor, checkpoint signaling, clock genes, p53, mathematical modeling, systematic approach

## Abstract

Circadian rhythms form a self-sustaining, endogenous, time-keeping system that allows organisms to anticipate daily environmental changes. The core of the clock network consists of interlocking transcriptional-translational feedback loops that ensures that metabolic, behavioral, and physiological processes run on a 24 h timescale. The hierarchical nature of the clock manifests itself in multiple points of control on the daily cell division cycle, which relies on synthesis, degradation, and post-translational modification for progression. This relationship is particularly important for understanding the role of clock components in sensing stress conditions and triggering checkpoint signals that stop cell cycle progression. A case in point is the interplay among the circadian factor PERIOD2 (PER2), the tumor suppressor p53, and the oncogenic mouse double minute-2 homolog protein (MDM2), which is the p53’s negative regulator. Under unstressed conditions, PER2 and p53 form a stable complex in the cytosol and, along with MDM2, a trimeric complex in the nucleus. Association of PER2 to the C-terminus end of p53 prevents MDM2-mediated ubiquitylation and degradation of p53 as well as p53’s transcriptional activation. Remarkably, when not bound to p53, PER2 acts as substrate for the E3-ligase activity of MDM2; thus, PER2 is degraded in a phosphorylation-independent fashion. Unexpectedly, the phase relationship between PER2 and p53 are opposite; however, a systematic modeling approach, inferred from the oscillatory time course data of PER2 and p53, aided in identifying additional regulatory scenarios that explained, *a priori*, seemingly conflicting experimental data. Therefore, we advocate for a combined experimental/mathematical approach to elucidating multilevel regulatory cellular processes.

## Introduction

Circadian oscillators provide living organisms an adaptive advantage by enabling them to anticipate the demands of an evolving environment. To accomplish this, organisms synchronize their metabolism and behavior to external cues through a core molecular mechanism, named the circadian clock, that is driven by interlocking transcriptional-translational feedback loops and is linked to regulatory output pathways (Bell-Pedersen et al., 2005). Briefly, the mammalian core clock system is sustained by three interlocking mechanisms that are controlled by a combination of timely distributed factors whose binding and dissociation to the gene’s regulatory regions dictate its phase of expression (for review see Takahashi, 2017 and references within). At the center of the network is the heterodimeric complex formed by the basic helix-loop-helix (bHLH)-PAR-ARNT-SIM (PAS) transcription factors CLOCK (circadian locomotor output cycles kaput and its paralog the Neuronal PAS domain Protein 2 NPAS2) and BMAL1 (Brain and Muscle ARNT-like 1 or the Aryl hydrocarbon Receptor Nuclear Translocator-Like protein 1 ARNTL). The CLOCK:BMAL1 complex binds to CAC(G/A)TG response elements (E-box, enhancer box) and activates the expression of *PERIOD* 1-3 (*PER1*, *PER2*, *PER3*) and *CRYPTOCHROME* 1-2 (*CRY1*, *CRY2*) genes. PER and CRY proteins accumulate asymmetrically during the day in the cytosol. PERs are targeted by casein kinases 1δ/ε (CK1δ/ε), phosphorylated, and degraded by the proteasome until the subsequent accumulation of CRY, later in the day, favors the formation of the PER:CRY:CK1δ/ε complex. This complex then shuttles to the nucleus where it interacts with CLOCK:BMAL1 to repress the transcription of *PER* and *CRY* genes. As *de novo* synthesis of PER and CRY molecules falls and the PER:CRY:CK1δ/ε complex is degraded, repression is released and CLOCK:BMAL1 becomes available for a new round of transcription. The CLOCK:BMAL1 complex also controls a second regulatory loop as it activates the expression of the *NR1D1* and *NR1D2* genes encoding the nuclear receptors REV-ERBα and β, respectively. The REV proteins compete with RORα/β/γ for RORE binding elements within the *BMAL1* promoter leading to repression or activation of *BMAL1*, respectively, which is antiphase to that of the *PER* genes. In a third regulatory loop, CLOCK:BMAL1 transcriptionally activates the expression of *DBP* (D-box binding PAR bZIP transcription factor) whose protein product binds to D-box response elements found in the regulatory regions of core clock components. Regardless, we have departed from the long-held belief that global rhythms of mRNA expression were exclusively driven by *de novo* rhythms in transcription and have embraced the contribution of post-transcriptional regulatory mechanisms in generating cycling messengers (Koike et al., 2012).

### Coupling Between the Circadian Clock and the Cell Division Cycle

Like the self-sustained and cell-autonomous circadian clock, mammalian cells undergo periodic oscillations in the form of successive rounds of division. Proliferative cells progress through the cell cycle in a process whose completion takes about a day. In a manner resembling the circadian clock, phase transitions, from Gs to S and M and back in the cell cycle, are driven by *de novo* transcription, protein accumulation, post-translational modifications, and protein degradation. As a result, the existence of a coupling mechanism between the two oscillators was initially described (Brown, 1991; Bjarnason et al., 1999, 2001) and later proven to exist in organisms as diverse as the prokaryotic cyanobacterium *Synechococcus* PCC 7942 (Mori et al., 1996; Yang et al., 2010), the fungi *Neurospora crassa* (Hong et al., 2014), zebrafish (Tamai et al., 2012), and mammalian cells (Matsuo et al., 2003; Nagoshi et al., 2004; Kowalska et al., 2013). Later work further refined the circadian-cell cycle relationship to establish temporal windows in the circadian cycle at which specific cell cycle transitions were more likely to occur (for review see Gaucher et al., 2018). This phenomenon, usually referred to as “*circadian gating of the cell cycle*,” has been proposed to provide a fitness benefit by, for example, ensuring that DNA replication takes place at times in which genotoxic stress is at minimum and metabolic conditions favor low levels of free radicals (Destici et al., 2011).

Of the many players involved in cell cycle progression, several key regulatory factors are directly controlled by circadian proteins. In a landmark study by Matsuo et al. (2003), the authors established that CLOCK:BMAL1 regulates the oscillatory expression of *Wee1*, a gene whose kinase product modulates the activity of cyclin B/Cdc2 and, therefore, G2/M progression in regenerating mouse liver. Accordingly, this mitotic clock-dependent gate was found to be disrupted in *cry1* deficient mice (Matsuo et al., 2003). Circadian factors also exert control over G1/S progression by transcriptionally modulating *Cdkn1a*, a gene that encodes for the cyclin/Cdk inhibitor p21^WAF1/CIP1^ (Grechez-Cassiau et al., 2008). Biochemical data backs the findings that clock orphan nuclear receptors REV-ERBα/β and RORα/γ exhibit antagonistic activities over *Cdkn1a* whereas genetic studies establish that their control is mediated by *Bmal1* (Grechez-Cassiau et al., 2008). A different G1 inhibitor, p16^ink4a^, which binds Cdk4/6 when it is dissociated from their cyclin counterpart, is also targeted for circadian regulation in primary fibroblasts (Kowalska et al., 2013). Accordingly, the complex between the nuclear RNA-binding protein NONO (non-POU domain-containing octamer-binding protein) and the circadian factor PER1 (NONO:PER1) drives the expression of *Cdkn2a* and, thus, the production of p16^ink4a^ in a manner that resembles PER1 expression (Kowalska et al., 2013). In fact, downregulation of NONO resulted in a non-rhythmic, low level expression of *Cdkn2a* and deregulation of G1 progression at a specific circadian phase (Kowalska et al., 2013). An additional key player in the process of cell growth and proliferation, the *c-Myc* oncogene is post-translationally regulated by CRY2 and the SCF^FBXL3^ ubiquitin ligase complex (Huber et al., 2016). In this scenario, CRY2-dependent turnover of MYC relies on MYC’s phosphorylation and its recognition by SCF^FBXL3^ in a process in which CRY2 seems to act as an adaptor/chaperone/presenter molecule for the E3 ubiquitin complex (Huber et al., 2016). Studies in more complex and heterogenous multicellular system, i.e., enteroids, established circadian gating of cell division is mediated by intercellular signals arising from differentiated cells (Matsu-Ura et al., 2016). Consequently, multi-level regulatory mechanisms seem to ensure that gating occurs at specific windows of time in a tightly regulated process.

Checkpoint mechanisms ensure the faithful completion of each discrete phase of the cell cycle before the next one proceeds. Checkpoints monitor, for example, that DNA duplication is completed, that accurate chromosome segregation occurred, and that the integrity of the genome remains intact. When considering the relevance of these processes in the context of “*timely progression*” through each cell cycle phase, it seems reasonable to speculate that clock mechanisms and checkpoint pathways would likely intersect. Today, evidence shows that crosstalk exists and occurs at multiple signaling levels; however, few studies explored the functional consequences beyond a descriptive level of analysis. For example, when PER1 is ectopically expressed, the checkpoint kinases Chk2 and ataxia-telangiectasia mutated (ATM) were found to co-immunoprecipitate with PER1 (Gery et al., 2006). Similarly, overexpression of PER1downregulated the levels of *Wee1*, *CcnB1* (encodes cyclin B1), and *Cdk1* (encodes Cdc2) mRNA and suppressed growth in cultured cancer cells (Gery et al., 2006). Another example is the product of human *Timeless* (hTIM), a gene with a well-established function in the *Drosophila* clock system (dTim) but with a debatable circadian role in mammalian cells (Mazzoccoli et al., 2016). Indeed, hTIM has little functional or structural resemblance to the dTim ortholog involved in clock function. It has a closer connection to dTim2, a molecule with a yet unclear role in circadian rhythms but which has relevance in DNA metabolism and chromosomal stability and integrity (Mazzoccoli et al., 2016). In this context, hTIM has been shown to bind to CRY2 and, in response to damage and replication stress, to CHK1 (Unsal-Kacmaz et al., 2005). Undoubtedly, one of the best-established connections between circadian components and checkpoint signaling arises from the findings that CRY1 and CRY2 have distinct roles in maintaining the integrity of the genome in response to genotoxic stress (Papp et al., 2015). In mammalian cells, CRY1 is phosphorylated and deubiquitylated by the Herpes virus associated ubiquitin-specific protease (HAUSP/Usp7) while CRY2 is destabilized through its interaction with SCF^FBXL3^ in response to DNA-damage (Papp et al., 2015). This counter effect on CRY proteins tilt their expression balance and, therefore, influences the targeted transcription of downstream genes in a manner that protects genomic stability (Papp et al., 2015).

More recently, we reported that the circadian factor PER2 directly binds to the tumor suppressor and G1/S checkpoint regulator p53 as well as its negative regulator, the oncogenic mouse double minute-2 homolog protein (MDM2) (Gotoh et al., 2014, 2015). Multiple layers of regulation connect MDM2 function with p53 stability and subcellular localization under normal conditions and in response to genotoxic stress. Briefly, binding of MDM2 to the N-terminal transactivation domain in p53 promotes either monoubiquitylation and nuclear export of p53 or polyubiquitylation and degradation by the 26S proteasome when p53 localizes in the cytosol (Kruse and Gu, 2009). As is the case with other post-translational modifications, ubiquitylation is a reversible process that, in the case of p53, is mediated by the deubiquitylating enzyme herpes virus-associated ubiquitin-specific protease (HAUSP, for review see Kruse and Gu, 2009).

Identification of p53 as a direct interactor of PER2 was first reported using a two-hybrid bacterial screening, confirmed by immunoprecipitation of the endogenous PER2:p53 complex, and proven by *in vitro* competition assays in various cell lines (Gotoh et al., 2014). Binding of PER2 occurs in p53’s C-terminus domain, which encompasses nuclear localization signals, both export and import, and the tetramerization domain required for p53 oligomerization and transcriptional activity (Gotoh et al., 2014). Interestingly, binding of PER2 to p53 does not preclude MDM2 recruitment to p53’s N-terminus domain and the existence of an endogenous MDM2:p53:PER2 trimeric complex was confirmed (Gotoh et al., 2014). From a functional standpoint, formation of the trimeric complex prevents MDM2-mediated ubiquitylation of p53 (Gotoh et al., 2014). This observation resulted from studies in which p53 ubiquitylation reactions were reconstituted in the presence of PER2 and from purified components *in vitro* (Gotoh et al., 2014). In others, p53’s half-life was estimated from cells where the endogenous level of PER2 was either up- or down-regulated (Gotoh et al., 2014). As a result, an initial model suggested that PER2 binding to p53 ensures that basal levels of the tumor suppressor exist for an acute response to, for example, genotoxic stress.

Subcellular distribution of PER2, p53, and MDM2 showed that whereas PER2:p53 localizes both in the cytosol and the nuclear compartments, MDM2:p53:PER2 complex remained solely in the nucleus (Gotoh et al., 2015). Gene expression studies have shown that dissociation of PER2 from p53 in the nucleus is an obligated step for p53 transcriptional activation (Gotoh et al., 2014, 2015). Accordingly, a chimera protein in which PER2 and p53 were covalently linked and folded was unable to modulate the expression of p53 downstream target genes (e.g., *SFN*, *BAX*, *CDKN1a*, *GADD45a*) (Gotoh et al., 2015). Furthermore, the effect of the chimera was achieved even in response to radiation and without compromising the upstream activation of the checkpoint response, i.e., the phosphorylation of ATM/ATR and CHK1/2 (Gotoh et al., 2015). In a related topic of yet unknown biological significance, overexpression of PER2 was shown to promote p53 transcription (Gotoh et al., 2014). Interestingly, the converse regulation in which p53 transcriptionally controls *PER2* gene expression has also been reported and linked to circadian behavior in animals (Miki et al., 2013).

**FIGURE 1:**
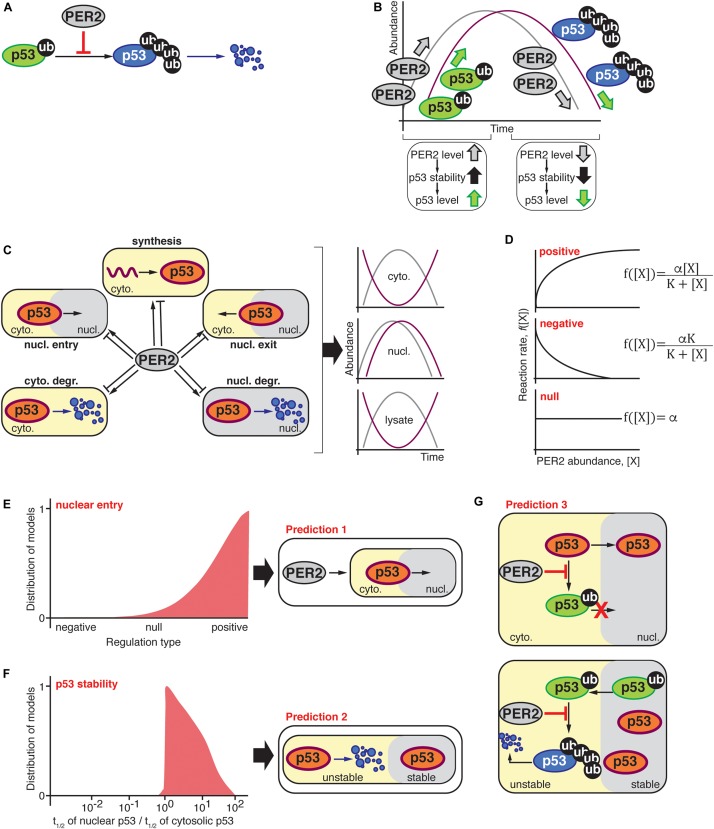
**(A)** PER2 inhibits MDM2-mediated polyubiquitylation of p53 resulting in p53 stabilization (Gotoh et al., 2014); thus, the abundance of PER2 and p53 were expected to oscillate in-phase **(B)**. **(C)** Schematic representation of possible scenarios by which PER2 could influence p53’s phase, i.e., p53 synthesis, shuttling, and degradation in either the cytosol or nuclear compartments. The oscillatory behavior of PER2 and p53 was significantly out-of-phase in both lysate and cytosolic fractions; but in-phase in the nucleus. **(D)** PER2-mediated regulation types for p53 synthesis, shuttling, and degradation were randomly selected among positive, negative, or null in agreement with experimentally determined PER2 and p53 phase relationships during the process of fitting (Gotoh et al., 2016). **(E,F)** Distribution of 10^3^ models that successfully simulated the phase relationship between p53 and PER2. The distribution of models is positively skewed toward a PER2-mediated regulation of p53 nuclear entry **(E)**. In addition, simulated fractions of p53’s half-life in nuclear and cytosolic compartments resulted in a value larger than one and, thus, p53 was predicted to be more stable in the nuclear compartment **(F)**. Predictions **(E,F)** were incorporated into a comprehensive mathematical model that considered potential molecular mechanisms for PER2 and p53 interaction. Specifically, PER2 promotes p53 nuclear entry by preventing p53’s mono-ubiquitylation **(E)**. Once in the nucleus, p53 is mono-ubiquitinated and exits to the cytoplasm, where it is poly-ubiquitinated and degraded **(F)**. As a result, the comprehensive model predicted that PER2 binding to p53 would occur regardless of p53’s ubiquitylation status to simulate the phase relationship between p53 and PER2 **(G)**.

An unexpected observation resulted from the analysis of PER2 and p53 oscillatory behavior in total cell extracts (Gotoh et al., 2016). Based on the finding that PER2 binds and stabilizes p53 (Figure 1A; Gotoh et al., 2014, 2015), the conventional wisdom would have been that PER2 and p53 levels oscillate in phase (Figure 1B). Instead, PER2 and p53 were found largely out-of-phase relative to each other in cytosolic fractions and matching cell lysates but in-phase in the nucleus (Gotoh et al., 2016). These, *a priori*, paradoxical findings prompted the development of a unique combined theoretical and experimental approach to shed light on the potential scenarios that could reconcile the current experimental data.

### An Unbiased Systematic Modeling Approach

We first asked what type of regulation, if any, does PER2 exert over p53 that results in an out-of-phase relationship between these proteins? Which, among the many regulatory processes in the cell that impact p53’s production, destruction, localization, and function are modulated by PER2’s biology? The relevance of these questions coincides with the complexity of their potential answers as a number of combinatorial scenarios that arise from considering all possible interactions and regulations surpass the capabilities of mathematical modeling and high-performance computing facilities. Furthermore, finding an explanation to the out-of-phase paradox was seriously underdetermined by the limited data available -only a handful of oscillatory timeseries for PER2 and p53.

A breakthrough came out when we simplified the p53’s complex regulatory network. Accordingly, the hundreds of biochemical processes that modulate p53 activity were categorized under common themes including p53’s synthesis, nuclear entry, nuclear exit, cytosolic degradation, and nuclear degradation (Figure 1C). Thus, the earlier questions were reduced to a simplified problem that could be tackled using ordinary differential equations and *in silico* experiments. To simulate all possible scenarios by which PER2 could influence production, shuttling, or degradation of p53 that resulted in in-phase distribution of PER and p53 in the nucleus but anti-phase in the cytosol, we derived the following equation:

$$\frac{d\,\left[p\,53_{c}\right]}{d\,t}=f_{1}\,\left(\left[P\,e\,r_{n}\right]\right)-f_{2}\,\left(\left[P\,e\,r_{c}\right]\right)\,\left[p\,53_{c}\right]+f_{4}\,\left(\left[P\,e\,r_{n}\right]\right)\,\left[p\,53_{n}\right]\frac{d\,\left[p\,53_{c}\right]}{d\,t}\;-f_{3}\,\left(\left[P\,e\,r_{c}\right]\right)\,\frac{\left[p\,53_{c}\right]}{K_{\deg}+\left[p\,53_{c}\right]}$$

(1) $$\frac{d\,\left[p\,53_{n}\right]}{d\,t}=f_{2}\,\left(\left[P\,e\,r_{c}\right]\right)\,\left[p\,53_{c}\right]-f_{4}\,\left(\left[P\,e\,r_{n}\right]\right)\,\left[p\,53_{n}\right]\;-f_{5}\,\left(\left[P\,e\,r_{n}\right]\right)\,\frac{\left[p\,53_{n}\right]}{K_{\deg}+\left[p\,53_{n}\right]}$$

where [*p53*_*c*_] and [*Per*_*c*_], [*p53*_*n*_] and [*Per*_*n*_] represent the concentration of p53 and PER2 in the cytoplasm and nucleus, respectively. In addition, oscillations in [*Per*_*c*_] and [*Per*_*n*_] were simulated *via* the Kim and Forger model, a mathematical model of the intracellular mammalian circadian clock (Kim and Forger, 2012; Kim et al., 2014; Kim, 2016).

Unlike most mathematical models, the functions *f*_1_, *f*_2_, *f*_3_, *f*_4_, and *f*_5_ were not specified in Equation (1). Each function describes a type of regulation on the production, nuclear import, cytosolic degradation, nuclear export, and nuclear degradation of p53, respectively, that is mediated by PER2 (Figure 1D). Regulation types were randomly selected among three function *f*_*i*_ forms:

(2) $$f_{i}\,\left(\left[X\right]\right)=\frac{\alpha_{i}\,\left[X\right]}{K_{i}+\left[X\right]},f_{i}\,\left(\left[X\right]\right)=\frac{\alpha_{i}\,K_{i}}{K_{i}+\left[X\right]},\text{or}\;f_{i}\,\left(\left[X\right]\right)=\alpha_{i}$$

which represent positive, negative, or null regulations *via* PER2 [[*X*] in Equation (2)], respectively. This approach allowed for the investigation of all possible scenarios for which PER2 mediates p53 regulation.

After unbiased selection of *f*_*i*_ functions (i.e., one-third for each regulation type of positive, null, or negative interactions), the value of the unknown parameters for these functions, i.e., *K*_*deg*_ in Equation (1) and *α_*i*_*, and *K*_*i*_ in Equation (2), were estimated using a global stochastic parameter search algorithm to simulate the phase relationship between PER2 and p53 (see Gotoh et al., 2016 for details). Of the ∼1 million simulated scenarios, 1,000 models successfully predicted the correct PER2 and p53 phase relationships in the cytosol and nucleus. Even when the selection of the regulation type was unbiased, successful cases showed a strong skewness toward a positive regulation of nuclear entry (Figure 1E). This implies that PER2-mediated p53 nuclear entry is essential to accurately simulate the proteins’ phase relationship (Prediction 1). Further analysis of the unknown parameter values (*α_*i*_*, and *K*_*i*_ and *K*_*deg*_) unveiled another unifying property among successful cases: p53 should be more stable in the nucleus than in the cytoplasm (Prediction 2, Figure 1F).

Predictions 1 and 2 arise from simplifying the biological system and neglecting detailed molecular mechanisms. Consequently, the model informs us about the “events,” e.g., PER2 shuttles p53 to the nucleus or p53 stability is altered in different compartments, but not about how the events are driven. For example, how does PER2 promote p53 nuclear entry? Or how is p53 stability achieved? Answers to the “hows” were arrived at by incorporating two key pieces of information from previous studies: (*i*) PER2 binding to p53 inhibits p53’s ubiquitylation (Figure 1A; Gotoh et al., 2014), and (*ii*) p53 ubiquitylation status modulates its shuttling and degradation (O’Brate and Giannakakou, 2003; Gotoh et al., 2016). By integrating both the latest information and the model’s predictions, a more detailed and realistic mathematical model emerged. As a result, a newly refined model included the PER transcriptional negative feedback loop and considered the various ubiquitylated forms of p53 along with the interaction between PER2 and p53; all of which, were summarized in 13 differential equations containing 18 parameters that were thoroughly described in Gotoh et al. (2016).

Two assumptions were at the foundation of the newly refined model: (*i*) PER2 promotes p53’s nuclear entry by blocking p53’s ubiquitylation within the nuclear import motif (Marchenko et al., 2010) and, (*ii*) once in the cytoplasm, mono-ubiquitylated p53 becomes poly-ubiquitylated and thus, unstable (Figure 1G; Marchenko et al., 2010). Along with restrictions in the parameters based on the aforementioned assumptions, parameters were estimated by the simulated annealing method. This allowed for the refined model to simulate the phase relationship between the two proteins. Following the analysis of the estimated parameters, we found that PER2:p53 binding should occur, regardless of p53’s ubiquitylation status, to properly simulate the antiphase relationship between PER2 and p53 that was reported to exist in total cell lysates (Prediction 3).

### From *in silico* Prediction to *in vitro* Validation

The three *in silico* predictions were next challenged in a cell-based system (Gotoh et al., 2016). Accordingly, experiments were devoted to test the prediction that PER2 binding to p53 promotes p53’s nuclear translocation (Figure 1E; prediction 1). The rationale for an experimental design was conceptually simple: If PER2 were to favor p53 translocation, then PER2 ectopic expression, or its downregulation, should impact the accumulation of p53 in either cellular compartment. In accordance with this premise, PER-mediated nuclear shuttling of p53 was confirmed in cells overexpressing trace levels of PER2 and maintained in the presence of both proteasome and nuclear export inhibitors (Gotoh et al., 2016). The converse experiment, in which endogenous PER2 was downregulated, also resulted in a reduction in the total level of p53 present in the nucleus but not its complete abrogation (Gotoh et al., 2016), suggesting that PER2 certainly contributes to p53 shuttling but that other routes exist.

Other work evaluated the half-life of p53 in purified nuclear and cytosolic extracts obtained from cells treated with cycloheximide, a protein translation inhibitor. In agreement with the theoretical model (Figure 1F; prediction 2), the half-life of endogenous p53 was ∼sevenfold longer when localized in the nucleus than in the cytosolic compartment and, thus, p53’s localization influences the kinetics of its degradation (Gotoh et al., 2016). Interestingly, PER2’s half-life remained comparable in both compartments.

Gotoh et al. (2014) established that PER2 binding to p53 prevents p53’s polyubiquitylation favoring its stability. This finding posed a provocative prediction that PER2 should be, nevertheless, able to bind a form of p53 containing multiple ubiquitin moieties (Figure 1G; prediction 3). Stepwise *in vitro* assays using recombinant purified proteins showed that, indeed, PER2 binds to mono and polyubiquitylated p53 (although, the extent to which the various forms of PER2:p53 complexes interplay remains to be explored) (Gotoh et al., 2016). As a result, the systematic mathematical approach helped to reconcile seemingly contradictory experimental data by generating theoretical predictions that were, then, experimentally confirmed.

### A Bidirectional Relationship Between Clock and Checkpoint Components

The interaction of PER2 with p53 and MDM2 brings to our attention the need for understanding the role that circadian proteins play at critical nodes of regulation in the cell, e.g., at the checkpoint crosstalk. Conversely, it poses the question of whether cellular p53 and/or MDM2 components influence the clock itself.

In eukaryotic organisms, the stability of PER2, which is responsive to environmental signals and homeostatic cellular conditions, is driven by multiple phosphorylation events that influence the period length and phase of the circadian rhythm (Gallego and Virshup, 2007; Chiu et al., 2011; Lee et al., 2011; Reischl and Kramer, 2011; D’Alessandro et al., 2015; Zhou et al., 2015). PER2 is targeted for post-translational modification by CK1ε/δ, glycogen synthase kinase 3β (GSK3β), casein kinase 2 (CK2), and casein kinase 1α (CK1α), all of which lead to either PER2 translocation or altered stability (for review see Ode and Ueda, 2018). Support for the role of CK1ε/δ in period determination emerged from multiple sources including genetic/phenotypic screenings in organisms as diverse as flies and mice, high-throughput studies using either small molecule inhibitors or short interfering RNAs, and disease genotyping in the human population (Kloss et al., 1998; Price et al., 1998; Lowrey et al., 2000; Xu et al., 2005; Badura et al., 2007; Hirota et al., 2008; Zhang et al., 2009; Meng et al., 2010; Chen et al., 2012; Kim et al., 2013, 2019). In the case of CK2, the kinase seems to play a dual role by phosphorylating the N-terminus domain of PER2 and by cooperating with CK1ε to promote PER2 degradation in the mammalian clock (Tsuchiya et al., 2009). Unlike CK1, the activity of GSK3β influences period length by targeting PER2 and promoting PER2’s nuclear translocation (Iitaka et al., 2005). Thus, PER2’s stability, shuttling, and the activity of various kinases are all part of an entangled network involved in period determination.

Key molecular events define the best understood mechanism for PER2 turnover; these include, CK1ε/δ substrate phosphorylation followed by the E3 ligase β-transducin repeat-containing protein 1/2 (β-TrCP1/2) recognition of PER2, ubiquitylation, and PER2’s proteasomal degradation (Eide et al., 2005; Ohsaki et al., 2008). However, a number of experimental observations suggested that additional mechanisms might exist. For example, co-expression of dominant negative forms of β-TrCP1 and β-TrCP2 in cells stabilize PER2 rather than promote its degradation (Ohsaki et al., 2008). Results reported by Walton et al. (2009) and Lee et al. (2011). were in the same line in which neither the expression of a dominant negative form of the CK1ε isoform in a CK1δ^–/–^ cellular background nor the pharmacological inhibition of CK1δ/ε isoforms, respectively, completely abrogated the circadian oscillation of a reporter gene. More recently, Zhou et al. proposed a phosphoswitch model to explain the three-stage kinetic of PER2 degradation that occurs during the PER2’s accumulation phase (Zhou et al., 2015; Narasimamurthy et al., 2018). Unlike the rapid initial decay and second accumulation stage, PER2’s degradation in both the third stage and falling phase of the circadian oscillation were independent of phosphorylation and β-TrCP activity (Zhou et al., 2015). Finally, ubiquitylated PER proteins were also detected in β-TrCP1/2 knockdown cells, although PER degradation occurred at a slower rate than in wild type cells (D’Alessandro et al., 2017). The above cumulative evidence suggests that a phosphorylation-independent and ubiquityl-mediated turnover of PER2 could exist. Thus, a role for MDM2 in modulating PER2 stability is plausible.

Identification of PER2 as a previously uncharacterized substrate of MDM2 was shown to occur in the absence of p53 binding (Liu et al., 2018). First, PER2:MDM2 association was detected by protein complementation and later confirmed by immunoprecipitation of the endogenous complex in various cellular systems (Liu et al., 2018). Conformational epitope mapping showed that PER2 binding occurs downstream of the p53-binding domain and upstream of the RING domain in MDM2 (Liu et al., 2018). This finding is of relevance when considering the spatial assembly of the PER2:p53:MDM2 complex in a context in which structural information of either component alone or in association with each other is missing. By binding downstream of the p53-binding domain, PER2 association to MDM2 makes it possible for p53 to bind to, and be part of, the PER2:p53:MDM2 complex as it was initially shown to exist in the nucleus (Gotoh et al., 2015). When PER2 bound to the N-terminus of the RING-domain, MDM2 was still able to exert its RING-dependent ubiquitin ligase activity toward its substrates. Accordingly, MDM2-mediated ubiquitylation of PER2 was shown to occur and be preferentially mediated by UbcH5a at the ubiquitin linkages Lys^11^ and Lys^48^ (Liu et al., 2018). Interestingly, polyubiquitylation sites in PER2 mapped within the interface of binding between PER2 and p53, a result that suggests PER2:p53 association might serve the purpose of stabilizing both components of the complex (Liu et al., 2018). Unlike the case of β-TrCP, where substrate phosphorylation was established to be an absolute requirement for β-TrCP recognition and ligase activity, phosphorylation of PER2 was neither a pre-requisite for MDM2 binding nor for MDM2-mediated ubiquitylation as shown in cultured cells and in a recombinant system (Liu et al., 2018). Corresponding with the role of polyubiquitylation signals in proteasomal degradation, the activity of MDM2 toward PER2 shortened PER2’s half-life whereas MDM2 down-regulation or its chemical inhibition favors PER2 stability (Liu et al., 2018). Accordingly, ectopic expression of MDM2 shortened the period length where its downregulation, or inhibition, resulted in a significant lengthening of the circadian period, which suggests that control over MDM2 activity is a relevant node of circadian regulation (Liu et al., 2018).

**FIGURE 2:**
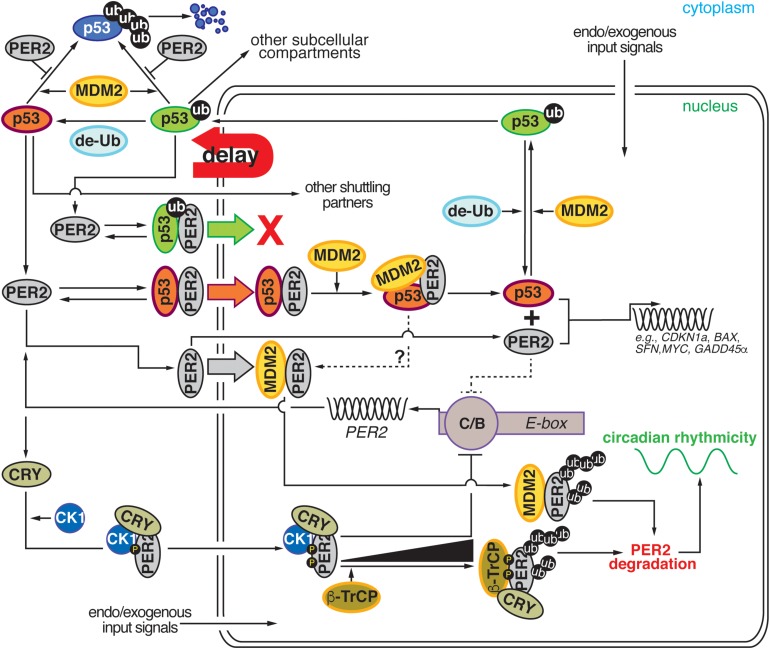
Comprehensive diagram representing the existing bidirectional interaction within the PER2:p53:MDM2 network. Cartoon representation depicting the four major regulatory nodes in p53, PER2, and MDM2 signaling. Node 1: PER2 modulates p53 stability by preventing MDM2-mediated ubiquitylation and proteasomal degradation of p53. Node 2: PER2:p53 binding favors p53 shuttling to the nucleus where it binds MDM2 and forms a stable trimeric complex. Node 3: Dissociation of PER2:p53 facilitates each component’s transcriptional activity and expression of downstream target genes. Node 4: Accumulation of PER2:MDM2 in the nucleus results in PER2 polyubiquitylation and degradation in a process that influences circadian period length. de-Ub, de-ubiquitylase; ub, ubiquitin; CK1δ/ε, casein kinase 1 δ/ε; CRY, cryptochrome; β-TrCP, β-transducin repeat-containing protein; PER2, period 2; MDM2, mouse double minute-2 homolog protein; C/B, circadian locomotor output cycles kaput/BMAL1 (Brain and Muscle ARNT-like (CLOCK/BMAL1); E-box, Enhancer element; g-IR, irradiation. Delay: represents the phase delay that results from PER2-mediated p53 shuttling.

The interplay between p53, MDM2, and PER2 establishes a node of regulation where circadian and checkpoint components can bidirectionally communicate and influence each other’s downstream signaling. As depicted in Figure 2, network connections can be clustered around five emerging themes: (*i*) PER2’s control of p53 stability, (*ii*) PER2’s regulation of nuclear p53 shuttling, (*iii*) PER2’s tuning of p53’s transcriptional activity, (*iv*) PER2’s interaction with MDM2, and (*v*) MDM2’s targeting of PER2 for degradation. All of these themes should be evaluated in the context of the well-established regulatory relationship between p53 and MDM2 (for review see Kruse and Gu, 2009; Vousden and Prives, 2009). In this relationship (*i*) p53 and MDM2 interact with each other, (*ii*) p53 protein levels oscillate in the cell, (*iii*) MDM2 mono-ubiquitylates p53 in the nucleus, (*iv*) the mono-ubiquitylated complex translocates to the cytosol where (*v*) p53 is polyubiquitiylated, and (*vi*) degraded by the proteasome. As a result, the picture that emerges is one in which a bidirectional regulation exists and occurs at multiple levels to ensure that events take place at specific times in the day and that enough time is given to the cell to respond to environmental and intracellular perturbations (Figure 2).

## Concluding Remarks

This work established that molecular connections between circadian and cell cycle checkpoint components occur bidirectionally. Quality research, as well as in-depth biochemical, molecular, structural, and behavioral studies in animal models aided by innovative modeling strategies, has helped in identifying previously unexpected crosstalk regulatory processes. This leaves us with the opportunity to tackle fundamental questions whose answers would certainly carry weight in the translational arena. For example, as circadian proteins interact with oncogene and tumor suppressor proteins, would the response to genotoxic stress be different in different circadian phases? Should therapeutic approaches aimed at targeting p53 be administered at specific times of the day? Is circadian rhythm disruption in response to DNA damage the consequence of changes in MDM2’s activity and, potentially, PER2’s stability? Answers to these questions lay in adequately retrieving system-level information and extending the complexity of existing models, an achievable goal with societal benefits in times where interdisciplinary research is being fostered.

## Author Contributions

XZ, DK, TG, and JL provided feedback and participated in the design of the figures. JK and CF wrote the manuscript and edited the figures. XZ, TG, JL, JK, and CF participated in the original research presented in the manuscript. All authors have read and approved the final version of this manuscript.

## Conflict of Interest

The authors declare that the research was conducted in the absence of any commercial or financial relationships that could be construed as a potential conflict of interest.

## References

[B1] Badura L. Swanson T. Adamowicz W. Adams J. Cianfrogna J. Fisher K. (2007). An inhibitor of casein kinase I epsilon induces phase delays in circadian rhythms under free-running and entrained conditions. *J. Pharmacol. Exp. Ther.* 322 730–738. 10.1124/jpet.107.122846 17502429

[B2] Bell-Pedersen D. Cassone V. M. Earnest D. J. Golden S. S. Hardin P. E. Thomas T. L. (2005). Circadian rhythms from multiple oscillators: lessons from diverse organisms. *Nat. Rev. Genet.* 6 544–556. 10.1038/nrg1633 15951747 PMC2735866

[B3] Bjarnason G. A. Jordan R. C. Sothern R. B. (1999). Circadian variation in the expression of cell-cycle proteins in human oral epithelium. *Am. J. Pathol.* 154 613–622. 10.1016/S0002-9440(10)65306-65300 10027418 PMC1849996

[B4] Bjarnason G. A. Jordan R. C. Wood P. A. Li Q. Lincoln D. W. Sothern R. B. (2001). Circadian expression of clock genes in human oral mucosa and skin: association with specific cell-cycle phases. *Am. J. Pathol.* 158 1793–1801. 10.1016/S0002-9440(10)64135-64131 11337377 PMC1891949

[B5] Brown W. R. (1991). A review and mathematical analysis of circadian rhythms in cell proliferation in mouse, rat, and human epidermis. *J. Invest. Dermatol.* 97 273–280. 10.1111/1523-1747.ep12480379 1830075

[B6] Chen Z. Yoo S. H. Park Y. S. Kim K. H. Wei S. Buhr E. (2012). Identification of diverse modulators of central and peripheral circadian clocks by high-throughput chemical screening. *Proc. Natl. Acad. Sci. U.S.A.* 109 101–106. 10.1073/pnas.1118034108 22184224 PMC3252927

[B7] Chiu J. C. Ko H. W. Edery I. (2011). NEMO/NLK phosphorylates PERIOD to initiate a time-delay phosphorylation circuit that sets circadian clock speed. *Cell* 145 357–370. 10.1016/j.cell.2011.04.002 21514639 PMC3092788

[B8] D’Alessandro M. Beesley S. Kim J. K. Chen R. Abich E. Cheng W. (2015). A tunable artificial circadian clock in clock-defective mice. *Nat. Commun.* 6:8587. 10.1038/ncomms9587 26617050 PMC4674671

[B9] D’Alessandro M. Beesley S. Kim J. K. Jones Z. Chen R. Wi J. (2017). Stability of wake-sleep cycles requires robust degradation of the PERIOD protein. *Curr. Biol.* 27 3454–3467.e8. 10.1016/j.cub.2017.10.014 29103939 PMC5698108

[B10] Destici E. Oklejewicz M. Saito S. van der Horst G. T. (2011). Mammalian cryptochromes impinge on cell cycle progression in a circadian clock-independent manner. *Cell Cycle* 10 3788–3797. 10.4161/cc.10.21.17974 22033214

[B11] Eide E. J. Woolf M. F. Kang H. Woolf P. Hurst W. Camacho F. (2005). Control of mammalian circadian rhythm by CKIepsilon-regulated proteasome-mediated PER2 degradation. *Mol. Cell Biol.* 25 2795–2807. 10.1128/MCB.25.7.2795-2807.2005 15767683 PMC1061645

[B12] Gallego M. Virshup D. M. (2007). Post-translational modifications regulate the ticking of the circadian clock. *Nat. Rev. Mol. Cell Biol.* 8 139–148. 10.1038/nrm2106 17245414

[B13] Gaucher J. Montellier E. Sassone-Corsi P. (2018). Molecular cogs: interplay between circadian clock and cell cycle. *Trends Cell Biol.* 28 368–379. 10.1016/j.tcb.2018.01.006 29471986

[B14] Gery S. Komatsu N. Baldjyan L. Yu A. Koo D. Koeffler H. P. (2006). The circadian gene per1 plays an important role in cell growth and DNA damage control in human cancer cells. *Mol. Cell* 22 375–382. 10.1016/j.molcel.2006.03.038 16678109

[B15] Gotoh T. Kim J. K. Liu J. Vila-Caballer M. Stauffer P. E. Tyson J. J. (2016). Model-driven experimental approach reveals the complex regulatory distribution of p53 by the circadian factor Period 2. *Proc. Natl. Acad. Sci. U.S.A.* 113 13516–13521. 10.1073/pnas.1607984113 27834218 PMC5127372

[B16] Gotoh T. Vila-Caballer M. Liu J. Schiffhauer S. Finkielstein C. V. (2015). Association of the circadian factor Period 2 to p53 influences p53’s function in DNA-damage signaling. *Mol. Biol. Cell* 26 359–372. 10.1091/mbc.E14-05-0994 25411341 PMC4294682

[B17] Gotoh T. Vila-Caballer M. Santos C. S. Liu J. Yang J. Finkielstein C. V. (2014). The circadian factor Period 2 modulates p53 stability and transcriptional activity in unstressed cells. *Mol. Biol. Cell* 25 3081–3093. 10.1091/mbc.E14-05-0993 25103245 PMC4230596

[B18] Grechez-Cassiau A. Rayet B. Guillaumond F. Teboul M. Delaunay F. (2008). The circadian clock component BMAL1 is a critical regulator of p21WAF1/CIP1 expression and hepatocyte proliferation. *J. Biol. Chem.* 283 4535–4542. 10.1074/jbc.M705576200 18086663

[B19] Hirota T. Lewis W. G. Liu A. C. Lee J. W. Schultz P. G. Kay S. A. (2008). A chemical biology approach reveals period shortening of the mammalian circadian clock by specific inhibition of GSK-3beta. *Proc. Natl. Acad. Sci. U.S.A.* 105 20746–20751. 10.1073/pnas.0811410106 19104043 PMC2606900

[B20] Hong C. I. Zamborszky J. Baek M. Labiscsak L. Ju K. Lee H. (2014). Circadian rhythms synchronize mitosis in *Neurospora crassa*. *Proc. Natl. Acad. Sci. U.S.A.* 111 1397–1402. 10.1073/pnas.1319399111 24474764 PMC3910635

[B21] Huber A. L. Papp S. J. Chan A. B. Henriksson E. Jordan S. D. Kriebs A. (2016). CRY2 and FBXL3 cooperatively degrade c-MYC. *Mol. Cell* 64 774–789. 10.1016/j.molcel.2016.10.012 27840026 PMC5123859

[B22] Iitaka C. Miyazaki K. Akaike T. Ishida N. (2005). A role for glycogen synthase kinase-3beta in the mammalian circadian clock. *J. Biol. Chem.* 280 29397–29402. 10.1074/jbc.M503526200 15972822

[B23] Kim D. W. Chang C. Chen X. Doran A. C. Gaudreault F. Wager T. (2019). Systems approach reveals photosensitivity and PER2 level as determinants of clock-modulator efficacy. *Mol. Syst. Biol.* 15:e8838. 10.15252/msb.20198838 31353796 PMC6613017

[B24] Kim J. K. (2016). Protein sequestration versus Hill-type repression in circadian clock models. *IET Syst. Biol.* 10 125–135. 10.1049/iet-syb.2015.0090 27444022 PMC8687308

[B25] Kim J. K. Forger D. B. (2012). A mechanism for robust circadian timekeeping via stoichiometric balance. *Mol. Syst. Biol.* 8:630. 10.1038/msb.2012.62 23212247 PMC3542529

[B26] Kim J. K. Forger D. B. Marconi M. Wood D. Doran A. Wager T. (2013). Modeling and validating chronic pharmacological manipulation of circadian rhythms. *CPT Pharmacomet. Syst. Pharmacol.* 2:e57. 10.1038/psp.2013.34 23863866 PMC3734602

[B27] Kim J. K. Josic K. Bennett M. R. (2014). The validity of quasi-steady-state approximations in discrete stochastic simulations. *Biophys. J.* 107 783–793. 10.1016/j.bpj.2014.06.012 25099817 PMC4129492

[B28] Kloss B. Price J. L. Saez L. Blau J. Rothenfluh A. Wesley C. S. (1998). The Drosophila clock gene double-time encodes a protein closely related to human casein kinase Iepsilon. *Cell* 94 97–107. 10.1016/s0092-8674(00)81225-81228 9674431

[B29] Koike N. Yoo S. H. Huang H. C. Kumar V. Lee C. Kim T. K. (2012). Transcriptional architecture and chromatin landscape of the core circadian clock in mammals. *Science* 338 349–354. 10.1126/science.1226339 22936566 PMC3694775

[B30] Kowalska E. Ripperger J. A. Hoegger D. C. Bruegger P. Buch T. Birchler T. (2013). NONO couples the circadian clock to the cell cycle. *Proc. Natl. Acad. Sci. U.S.A.* 110 1592–1599. 10.1073/pnas.1213317110 23267082 PMC3562797

[B31] Kruse J. P. Gu W. (2009). Modes of p53 regulation. *Cell* 137 609–622. 10.1016/j.cell.2009.04.050 19450511 PMC3737742

[B32] Lee H. M. Chen R. Kim H. Etchegaray J. P. Weaver D. R. Lee C. (2011). The period of the circadian oscillator is primarily determined by the balance between casein kinase 1 and protein phosphatase 1. *Proc. Natl. Acad. Sci. U.S.A.* 108 16451–16456. 10.1073/pnas.1107178108 21930935 PMC3182690

[B33] Liu J. Zou X. Gotoh T. Brown A. M. Jiang L. Wisdom E. L. (2018). Distinct control of PERIOD2 degradation and circadian rhythms by the oncoprotein and ubiquitin ligase MDM2. *Sci. Signal.* 11:556 10.1126/scisignal.aau0715 30425162

[B34] Lowrey P. L. Shimomura K. Antoch M. P. Yamazaki S. Zemenides P. D. Ralph M. R. (2000). Positional syntenic c. *Science* l 483–492. 10.1126/science.288.5465.483 PMC3869379 10775102

[B35] Marchenko N. D. Hanel W. Li D. Becker K. Reich N. Moll U. M. (2010). Stress-mediated nuclear stabilization of p53 is regulated by ubiquitination and importin-alpha3 binding. *Cell Death Differ.* 17 255–267. 10.1038/cdd.2009.173 19927155 PMC4419752

[B36] Matsuo T. Yamaguchi S. Mitsui S. Emi A. Shimoda F. Okamura H. (2003). Control mechanism of the circadian clock for timing of cell division in vivo. *Science* 302 255–259. 10.1126/science.1086271 12934012

[B37] Matsu-Ura T. Dovzhenok A. Aihara E. Rood J. Le H. Ren Y. (2016). Intercellular coupling of the cell cycle and circadian clock in adult stem cell culture. *Mol. Cell* 64 900–912. 10.1016/j.molcel.2016.10.015 27867006 PMC5423461

[B38] Mazzoccoli G. Laukkanen M. O. Vinciguerra M. Colangelo T. Colantuoni V. (2016). A timeless link between circadian patterns and disease. *Trends Mol. Med.* 22 68–81. 10.1016/j.molmed.2015.11.007 26691298

[B39] Meng Q. J. Maywood E. S. Bechtold D. A. Lu W. Q. Li J. Gibbs J. E. (2010). Entrainment of disrupted circadian behavior through inhibition of casein kinase 1 (CK1) enzymes. *Proc. Natl. Acad. Sci. U.S.A.* 107 15240–15245. 10.1073/pnas.1005101107 20696890 PMC2930590

[B40] Miki T. Matsumoto T. Zhao Z. Lee C. C. (2013). p53 regulates Period2 expression and the circadian clock. *Nat. Commun.* 4:2444. 10.1038/ncomms3444 24051492 PMC3798035

[B41] Mori T. Binder B. Johnson C. H. (1996). Circadian gating of cell division in cyanobacteria growing with average doubling times of less than 24 hours. *Proc. Natl. Acad. Sci. U.S.A.* 93 10183–10188. 10.1073/pnas.93.19.10183 8816773 PMC38358

[B42] Nagoshi E. Saini C. Bauer C. Laroche T. Naef F. Schibler U. (2004). Circadian gene expression in individual fibroblasts: cell-autonomous and self-sustained oscillators pass time to daughter cells. *Cell* 119 693–705. 10.1016/j.cell.2004.11.015 15550250

[B43] Narasimamurthy R. Hunt S. R. Lu Y. Fustin J. M. Okamura H. Partch C. L. (2018). CK1delta/epsilon protein kinase primes the PER2 circadian phosphoswitch. *Proc. Natl. Acad. Sci. U.S.A.* 115 5986–5991. 10.1073/pnas.1721076115 29784789 PMC6003379

[B44] O’Brate A. Giannakakou P. (2003). The importance of p53 location: nuclear or cytoplasmic zip code? *Drug Resis.t Updat.* 6 313–322. 10.1016/j.drup.2003.10.004 14744495

[B45] Ode K. L. Ueda H. R. (2018). Design principles of phosphorylation-dependent timekeeping in eukaryotic circadian clocks. *Cold Spring Harb. Perspect. Biol.* 10:a028357. 10.1101/cshperspect.a028357 29038116 PMC6071485

[B46] Ohsaki K. Oishi K. Kozono Y. Nakayama K. Nakayama K. I. Ishida N. (2008). The role of {beta}-TrCP1 and {beta}-TrCP2 in circadian rhythm generation by mediating degradation of clock protein PER2. *J. Biochem.* 144 609–618. 10.1093/jb/mvn112 18782782

[B47] Papp S. J. Huber A. L. Jordan S. D. Kriebs A. Nguyen M. Moresco J. J. (2015). DNA damage shifts circadian clock time via Hausp-dependent Cry1 stabilization. *eLife* 4:e04883. 10.7554/eLife.04883 25756610 PMC4352707

[B48] Price J. L. Blau J. Rothenfluh A. Abodeely M. Kloss B. Young M. W. (1998). double-time is a novel Drosophila clock gene that regulates PERIOD protein accumulation. *Cell* 94 83–95. 10.1016/s0092-8674(00)81224-81226 9674430

[B49] Reischl S. Kramer A. (2011). Kinases and phosphatases in the mammalian circadian clock. *FEBS Lett.* 585 1393–1399. 10.1016/j.febslet.2011.02.038 21376720

[B50] Takahashi J. S. (2017). Transcriptional architecture of the mammalian circadian clock. *Nat. Rev. Genet.* 18 164–179. 10.1038/nrg.2016.150 27990019 PMC5501165

[B51] Tamai T. K. Young L. C. Cox C. A. Whitmore D. (2012). Light acts on the zebrafish circadian clock to suppress rhythmic mitosis and cell proliferation. *J. Biol. Rhythms* 27 226–236. 10.1177/0748730412440861 22653891

[B52] Tsuchiya Y. Akashi M. Matsuda M. Goto K. Miyata Y. Node K. (2009). Involvement of the protein kinase CK2 in the regulation of mammalian circadian rhythms. *Sci. Signal.* 2:ra26. 10.1126/scisignal.2000305 19491384

[B53] Unsal-Kacmaz K. Mullen T. E. Kaufmann W. K. Sancar A. (2005). Coupling of human circadian and cell cycles by the timeless protein. *Mol. Cell. Biol.* 25 3109–3116. 10.1128/MCB.25.8.3109-3116.2005 15798197 PMC1069621

[B54] Vousden K. H. Prives C. (2009). Blinded by the light: the growing complexity of p53. *Cell* 137 413–431. 10.1016/j.cell.2009.04.037 19410540

[B55] Walton K. M. Fisher K. Rubitski D. Marconi M. Meng Q. J. Sladek M. (2009). Selective inhibition of casein kinase 1 epsilon minimally alters circadian clock period. *J. Pharmacol. Exp. Ther.* 330 430–439. 10.1124/jpet.109.151415 19458106

[B56] Xu Y. Padiath Q. S. Shapiro R. E. Jones C. R. Wu S. C. Saigoh N. (2005). Functional consequences of a CKIdelta mutation causing familial advanced sleep phase syndrome. *Nature* 434 640–644. 10.1038/nature03453 15800623

[B57] Yang Q. Pando B. F. Dong G. Golden S. S. van Oudenaarden A. (2010). Circadian gating of the cell cycle revealed in single cyanobacterial cells. *Science* 327 1522–1526. 10.1126/science.1181759 20299597 PMC3118046

[B58] Zhang E. E. Liu A. C. Hirota T. Miraglia L. J. Welch G. Pongsawakul P. Y. (2009). A genome-wide RNAi screen for modifiers of the circadian clock in human cells. *Cell* 139 199–210. 10.1016/j.cell.2009.08.031 19765810 PMC2777987

[B59] Zhou M. Kim J. K. Eng G. W. Forger D. B. Virshup D. M. (2015). A Period2 phosphoswitch regulates and temperature compensates circadian period. *Mol. Cell* 60 77–88. 10.1016/j.molcel.2015.08.022 26431025

